# SAELGMDA: Identifying human microbe–disease associations based on sparse autoencoder and LightGBM

**DOI:** 10.3389/fmicb.2023.1207209

**Published:** 2023-06-21

**Authors:** Feixiang Wang, Huandong Yang, Yan Wu, Lihong Peng, Xiaoling Li

**Affiliations:** ^1^School of Computer Science, Hunan University of Technology, Zhuzhou, China; ^2^Department of Gastrointestinal Surgery, Yidu Central Hospital of Weifang, Weifang, China; ^3^Geneis (Beijing) Co., Ltd., Beijing, China; ^4^The Second Department of Oncology, Beidahuang Industry Group General Hospital, Harbin, China; ^5^The Second Department of Oncology, Heilongjiang Second Cancer Hospital, Harbin, China

**Keywords:** microbe-disease association, feature representation, dimensional reduction, sparse autoencoder, LightGBM

## Abstract

**Introduction:**

Identification of complex associations between diseases and microbes is important to understand the pathogenesis of diseases and design therapeutic strategies. Biomedical experiment-based Microbe-Disease Association (MDA) detection methods are expensive, time-consuming, and laborious.

**Methods:**

Here, we developed a computational method called SAELGMDA for potential MDA prediction. First, microbe similarity and disease similarity are computed by integrating their functional similarity and Gaussian interaction profile kernel similarity. Second, one microbe-disease pair is presented as a feature vector by combining the microbe and disease similarity matrices. Next, the obtained feature vectors are mapped to a low-dimensional space based on a Sparse AutoEncoder. Finally, unknown microbe-disease pairs are classified based on Light Gradient boosting machine.

**Results:**

The proposed SAELGMDA method was compared with four state-of-the-art MDA methods (MNNMDA, GATMDA, NTSHMDA, and LRLSHMDA) under five-fold cross validations on diseases, microbes, and microbe-disease pairs on the HMDAD and Disbiome databases. The results show that SAELGMDA computed the best accuracy, Matthews correlation coefficient, AUC, and AUPR under the majority of conditions, outperforming the other four MDA prediction models. In particular, SAELGMDA obtained the best AUCs of 0.8358 and 0.9301 under cross validation on diseases, 0.9838 and 0.9293 under cross validation on microbes, and 0.9857 and 0.9358 under cross validation on microbe-disease pairs on the HMDAD and Disbiome databases. Colorectal cancer, inflammatory bowel disease, and lung cancer are diseases that severely threat human health. We used the proposed SAELGMDA method to find possible microbes for the three diseases. The results demonstrate that there are potential associations between *Clostridium coccoides* and colorectal cancer and one between Sphingomonadaceae and inflammatory bowel disease. In addition, *Veillonella* may associate with autism. The inferred MDAs need further validation.

**Conclusion:**

We anticipate that the proposed SAELGMDA method contributes to the identification of new MDAs.

## 1. Introduction

Human microbes are a class of organisms with simple structure and small size (Wu et al., 2018; Cheng et al., 2020). They widely distribute in various organs of the human body including the gut, gastrointestinal tract, lung, oral cavity, and skin (Lynch and Pedersen, 2016). Its abnormality may cause diseases, such as cancers, inflammatory bowel disease (El Mouzan et al., 2018), and asthma (Demirci et al., 2019). Therefore, it is important to uncover potential associations between microbes and diseases. Identification of Microbe-Disease Associations (MDAs) helps capture the complex pathogenesis of various diseases and provides novel insights into its drug design. For example, a few methods have been developed to capture potential drugs against COVID-19 (Peng et al., 2022a; Shen L. et al., 2022; Tian et al., 2022). Traditional experimental methods are expensive, time-consuming, and laborious (Chen et al., 2019). Thus, much attention has been devoted to computational methods for new MDA prediction.

Many computational models have been designed to find potential MDAs based on known MDAs and biological features of diseases and microbes. These methods mainly contain network-based methods and machine learning-based methods. Network-based MDA prediction methods include the KATZ measurement (Zhang et al., 2017; Li et al., 2019), random walk with network topology structure (NTSHMDA) (Luo and Long, 2018), and bi-random walk (Zou et al., 2017; Luo and Long, 2018; Yan et al., 2019). Network-based methods effectively found a few new MDAs; however, they depend on known MDAs for similarity calculation and fail to screen possible microbes (or diseases) for a new disease (or microbes) that has no association prediction.

Machine learning-based MDA prediction methods contain Laplacian regularized least squares (LRLSHMDA) (Wang et al., 2017), binary matrix completion (Shi et al., 2018), graph regularized non-negative matrix factorization (He et al., 2018), logistic matrix factorization with neighborhood regularization combining positive-unlabeled learning (Peng et al., 2020), inductive matrix completion and graph attention networks (GATMDA) (Long et al., 2021), and low-rank matrix completion combining the nuclear norm minimization (MNNMDA) (Liu H. et al., 2023). Machine learning algorithms better improved MDA prediction.

In particular, deep learning has been increasingly applied to the area of bioinformatics, such as cardiotoxicity identification related to hERG channel blockers (Wang T. et al., 2023), protein model quality assessment (Guo et al., 2022; Liu J. et al., 2023), metabolite-disease association discovery (Sun et al., 2022), lncRNA-protein interaction prediction (Lihong et al., 2021), lncRNA-miRNA association inference (Chen et al., 2021; Wang et al., 2022), lncRNA-disease association identification (Liang et al., 2022; Zhang et al., 2023), single-cell data analysis (Hu et al., 2023; Xu et al., 2023), drug-target interaction detection (Zhang et al., 2022; Li et al., 2023), and intercellular communication analyses (Peng et al., 2022b). Similarly, deep learning has been widely applied to accurate MDA prediction. These methods include deep matrix factorization combining Bayesian personalized ranking (Liu et al., 2020), multi-component graph attention network (Liu et al., 2021), graph convolutional network (Hua et al., 2022), metapath aggregated graph neural network (Chen and Lei, 2022), dual network contrastive learning model (Cheng et al., 2022), weighted meta-graph-based model (Long and Luo, 2019), knowledge graph neural network (Jiang et al., 2022), and relation graph convolutional network (Wang Y. et al., 2023).

Deep learning efficiently implements accurate MDA identification. In this manuscript, we developed a computational MDA prediction method called SAELGMDA by combining a sparse autoencoder for feature extraction and Light Gradient Boosting Machine (LightGBM) for MDA classification.

## 2. Materials and methods

### 2.1. Data description

To construct a human MDA network, we investigated a human MDA database called HMDAD provided by Ma et al. (2017) (http://www.cuilab.cn/hmdad). The database contains 483 experimentally confirmed MDAs between 39 diseases and 292 microbes. We finally achieved 450 MDAs after filtering repetitive MDAs. In addition, Janssens et al. (2018) have collected a new MDA database named Disbiome. The database contains 5,573 experimentally confirmed human MDAs between 1,098 microbes and 240 diseases. Finally, we obtained 4,351 MDAs between 1,052 microbes and 218 diseases after filtering repetitive MDAs.

Consequently, an element *X*_*ij*_ in an MDA matrix $X\in R^{n_{d}\times n_{m}}$ is represented as Eq. (1):


(1) $$X_{ij}=\;\{\begin{array}{cc} 1 & \text{ if disease }d_{i}\text{ associates with microbe }m_{j}\\ 0 & \text{ otherwise} \end{array}$$

where *n*_*d*_ and *n*_*m*_ indicate the number of diseases and microbes, respectively. An MDA is taken as a positive sample if *X*_*ij*_ = 1, otherwise, it is taken as an unlabeled sample.

### 2.2. Methods

**Figure 1:**
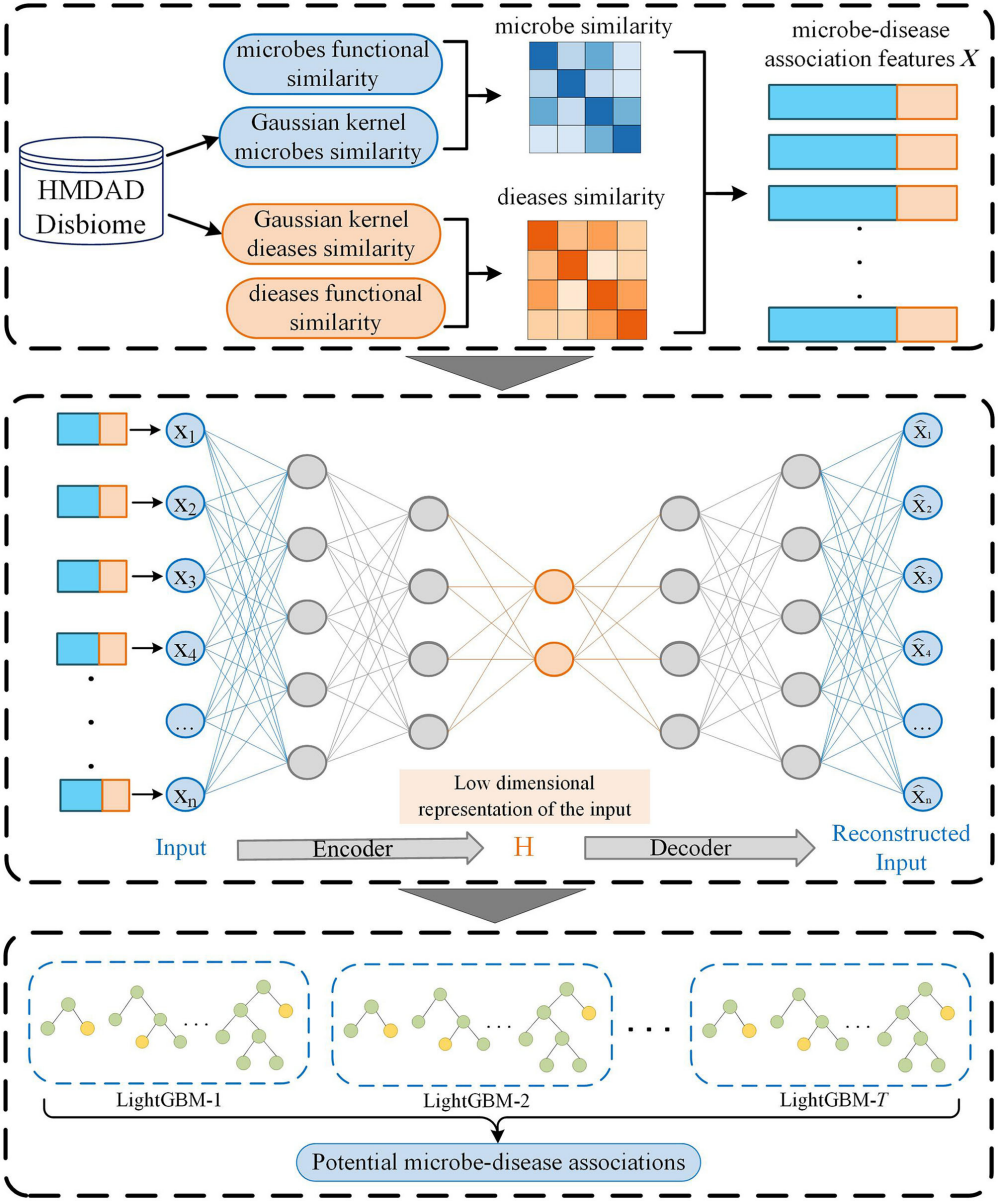
The flowchart of SAELGMDA.

In this manuscript, we proposed an MDA prediction method called SAELGMDA by combining sparse autoencoder and LightGBM. First, disease similarity and microbe similarity are computed by integrating functional similarity and Gaussian Interaction Profile Kernel (GIPK) similarity. Second, one microbe–disease pair is represented as one *d*-dimensional vector. Third, the obtained features for microbe–disease pairs are mapped into a low-dimensional space via a sparse autoencoder. Finally, the low-dimensional features are fed to LightGBM for MDA classification. The pipeline of SAELGBM is illustrated in Figure 1.

#### 2.2.1. Functional similarity of diseases and microbes

We considered that similar diseases are more likely to associate with similar genes (Xu and Li, 2006; Wei and Liu, 2020) and computed disease functional similarity via disease-related genes. For two diseases *d*_*i*_ and *d*_*j*_ and corresponding associated gene sets *G*_*i*_ = {*g*_*i*_1__, *g*_*i*_2__, …, *g*_*i*_*a*__} and *G*_*j*_ = {*g*_*j*_1__, *g*_*j*_2__, …, *g*_*j*_*b*__}, the functional association between gene *g*_*k*_ and gene set *G* = {*g*_1_, *g*_2_, …, *g*_*l*_} is first defined by Eq. (2):


(2) $$\begin{array}{l} F_{G}\left(g_{k}\right)=\underset{g_{t}\in G}{\max}\left(FS\left(g_{k},g_{t}\right)\right) \end{array}$$

where *FS*(*g*_*k*_, *g*_*t*_) indicates the functional similarity between *g*_*k*_ and *g*_*t*_ by Eq. (3):


(3) $$FS(g_{k},g_{t})=\{\begin{array}{cc} 1, & \text{ if }k=t\\ LLS^{\prime }\left(g_{k},g_{t}\right), & \text{ if }k\neq t \end{array}\;$$

where *LLS*′ denotes the normalized score of *LLS* by Eq. (4):


(4) $$\begin{array}{l} LLS^{\prime }\left(g_{k},g_{t}\right)=\frac{LLS\left(g_{k},g_{t}\right)-LLS_{\min}}{LLS_{\max}-LLS_{\min}} \end{array}$$

where *LLS* represents association log-likelihood score used to evaluate the functional linkage probability between two genes provided by HumanNet (Hwang et al., 2019; Long et al., 2021), *LLS*_max_ and *LLS*_min_ denote its maximum and minimum values, respectively.

Finally, the functional similarity between *d*_*i*_ and *d*_*j*_ is computed by Eq. (5):


(5) $$\begin{array}{l} D_{f}(d_{i},d_{j})=\frac{\sum_{g_{t}\in G(d_{i})}F_{G(d_{j})}(g_{t})+\sum_{g_{t}\in G(d_{j})}F_{G(d_{i})}(g_{t})}{a+b} \end{array}$$

Microbe functional similarity matrix *M*_*f*_ is computed based on the method proposed by Kamneva (2017).

#### 2.2.2. GIPK similarity of diseases and microbes

Based on the assumption that functionally similar diseases usually associate or disassociate with similar microbes, and disease Gaussian Interaction Profile Kernel (GIPK) similarity (Van Laarhoven et al., 2011) is computed via experimentally validated MDA network. In particular, the GIPK similarity of two diseases *d*_*i*_ and *d*_*j*_ is computed by Eq. (6):


(6) $$\begin{array}{l} D_{G}\left(d_{i},d_{j}\right)=\exp\left(-\gamma_{d}||IP\left(d_{i}\right)-IP\left(d_{j}\right)|{|}^{2}\right) \end{array}$$

where


(7) $$\begin{array}{l} \gamma_{d}=\gamma_{d}^{\prime }/\left(\frac{1}{n_{d}}\overset{n_{d}}{\underset{i=1}{\sum }}||IP\left(d_{i}\right)|{|}^{2}\right) \end{array}$$

and *IP*(*d*_*i*_) denotes associations between disease *d*_*i*_ and each microbe, that is, the *i*th row of *X*. γ_*d*_ denotes the normalized kernel bandwidth with original bandwidth $\gamma_{d}^{\prime }$ of 1, and *n*_*d*_ denotes the number of diseases.

Similarly, we computed the GIPK similarity matrix *M*_*G*_ of microbes.

#### 2.2.3. Similarity integration for diseases and microbes

We may fail to compute the functional similarity for all diseases because not all diseases have related to genes. Thus, we combined disease GIPK similarity and functional similarity by Eq. (8):


(8) $$S_{D}(d_{i},d_{j})=\{\begin{array}{cc} \frac{1}{2}(D_{f}(d_{i},d_{j})+D_{G}(d_{i},d_{j})) & \text{ if }D_{f}\left(d_{i},d_{j}\right)\neq 0\\ D_{f}\left(d_{i},d_{j}\right) & \text{ otherwise } \end{array}$$

Similarly, the integrated microbe similarity *S*_*M*_ is computed.

#### 2.2.4. Feature representation for microbe–disease associations

For each microbe–disease pair (*m*_*i*_, *d*_*j*_), feature vectors of *m*_*i*_ and *d*_*j*_ are obtained based on similarity matrices *S*_*D*_ and *S*_*M*_, respectively. Particularly, the feature vector of *d*_*i*_ is denoted as the similarity between *d*_*i*_ and all diseases. The feature vector of *m*_*j*_ is denoted as the similarity between *m*_*j*_ and all microbes. Thus, one microbe–disease pair is depicted as an (*n*_*d*_+*n*_*m*_)-dimensional feature vector after concatenation operation, where *n*_*d*_ and *n*_*m*_ indicate the number of diseases and microbes, respectively. In summary, there are *n*(*n* = *n*_*d*_×*n*_*m*_) samples (microbe–disease pairs), and each sample *x*_*i*_ can be represented using a *d*(*d* = *n*_*d*_+*n*_*m*_)-dimensional vector. For *x*_*i*_, its label *y*_*i*_ = 1. If its corresponding microbe–disease pair is associated, otherwise *y*_*i*_ = 0. Consequently, an MDA matrix *X* with *n* samples is represented by Eq. (9):


(9) $$\begin{array}{l} X={\left(\left[\begin{array}{c} S_{M_{1}}\\ S_{D_{1}} \end{array}\right],\cdots \;,\left[\begin{array}{c} S_{M_{1}}\\ S_{D_{n_{d}}} \end{array}\right],\cdots \;,\left[\begin{array}{c} S_{M_{n_{m}}}\\ S_{D_{1}} \end{array}\right],\cdots \;,\left[\begin{array}{c} S_{M_{n_{m}}}\\ S_{D_{n_{d}}} \end{array}\right]\right)}^{T} \end{array}$$

#### 2.2.5. Feature extraction based on sparse autoencoder

The obtained features for microbe–disease pairs are highly dimensional and severely affect the classification accuracy of models. Deep learning demonstrates stronger feature learning ability than traditional dimensional reduction approaches. Thus, we designed a sparse autoencoder to reduce the feature dimensionality of each sample.

Sparse autoencoder (Andrew, 2011) is an unsupervised neural network model. It minimizes the reconstruction error and enforces sparsity constraints on all hidden nodes to obtain a more robust and meaningful representation of features and further improves the prediction performance of classification models (Makhzani and Frey, 2013). First, a high-dimensional feature vector for the microbe–disease pair is fed to an encoder by Eq. (10):


(10) $$\begin{array}{l} H=f\left(WX+b\right) \end{array}$$

where *X* represents the input *n* samples with *d*-dimensional vector, *H* denotes the low-dimensional features after encoding, *W*, *b*, and *f*(·) represent the weight, bias, and encoding function of the encoder, respectively.

Next, a decoder restores the low-dimensional representation *H* to the same appearance as the input feature representation by Eq. (11):


(11) $$\begin{array}{l} \hat{X}=g\left(W^{\prime }H+b^{\prime }\right) \end{array}$$

where *W*′, *b*′, and *g*(·) represent the weight, bias, and decoding function of the decoder, respectively, and $\hat{X}$ denotes the learned feature representation.

To minimize the reconstruction error, we build a cost function by Eq. (12):


(12) $$\begin{array}{l} E=MSE+\lambda \times \Omega_{\text{sparsity}}+\beta \times \Omega_{\text{weights}} \end{array}$$

where λ and β denote the sparsity regularization parameter and the coefficients for *L*_2_ regularization, respectively.

The first term *MSE* is mean square error. The term is used to measure the discrepancy between the input features *X* and the reconstructed features $\hat{X}$ on training data by Eq. (13):


(13) $$\begin{array}{l} MSE=\frac{1}{n}\overset{n}{\underset{i=1}{\sum }}{\left(X_{i}-{\hat{X}}_{i}\right)}^{2} \end{array}$$

The second term Ω_sparsity_ is the Kullback–Leibler divergence. The term is used to control sparsity based on the sparsity proportion ρ by Eq. (14):


(14) $$\begin{array}{l} \Omega_{\text{sparsity}}=\overset{s_{l}}{\underset{t=1}{\sum }}KL\left(\rho ||{\hat{\rho }}_{t}\right) \end{array}$$

where *s*_*l*_ and ${\hat{\rho }}_{t}$ denote the number of neurons in the *l*th hidden layer and the average activity of the *t*th neuron, respectively, $KL\left(\rho ||\hat{\rho_{t}}\right)$ denotes the relative entropy between Bernoulli random variables with mean ρ and mean ${\hat{\rho }}_{t}$. $KL\left(\rho ||\hat{\rho_{t}}\right)$ is computed by Eq. (15):


(15) $$\begin{array}{l} KL\left(\rho ||\hat{\rho_{t}}\right)=\rho \log\frac{\rho }{{\hat{\rho }}_{t}}+\left(1-\rho \right)\log\frac{1-\rho }{1-{\hat{\rho }}_{t}} \end{array}$$

The third term is *L*_2_ regularization term Ω_weights_. The term is used to control the weights and avoid overfitting by Eq. (16):


(16) $$\begin{array}{l} \Omega_{\text{weights}}=\frac{1}{2}\overset{n_{l}-1}{\underset{l=1}{\sum }}\overset{s_{l}}{\underset{i=1}{\sum }}\overset{s_{l}+1}{\underset{j=1}{\sum }}{\left(w_{ji}^{\left(l\right)}\right)}^{2} \end{array}$$

where *n*_*l*_, *s*_*l*_, and $w_{ji}^{\left(l\right)}$ denote the number of layers, the number of units in the *l*th layer, and the weight, respectively.

#### 2.2.6. MDA classification based on LightGBM

Each microbe–disease pair is represented as a low-dimensional vector after dimensional reduction based on a sparse autoencoder. LightGBM (Ke et al., 2017) is an optimized version of Gradient Boosting Decision Tree (GBDT) (Ye et al., 2009). It obtains better performance in the area of bioinformatics. Next, the constructed low-dimensional vector is used as the input of LightGBM (Ke et al., 2017), to classify each microbe–disease pair. For an MDA dataset $D={\left\{\left(x_{i},y_{i}\right)\right\}}_{i=1}^{n}$, LightGBM intends to learn an approximation $\hat{f}$ to a certain function *f*(*x*) by minimizing the expectation of the loss function *L*(*y, f*(*x*)) by Eq. (17):


(17) $$\begin{array}{l} \hat{f}=\arg\underset{f}{\min}E_{x,y}\left[L\left(y,f\left(x\right)\right)\right] \end{array}$$

LightGBM integrates *T* decision trees $\overset{T}{\underset{t=1}{\sum }}f_{t}\left(X\right)$ to approximate the final model $f_{T}\left(X\right)=\overset{T}{\underset{t=1}{\sum }}f_{t}\left(X\right)$. The decision trees with *J* leaf nodes are expressed as *w*_*q*(*x*)_, where *w*_*q*(*x*)_ denotes the weights of all samples on leaf nodes and *q*(*x*) denotes the decision rules. Hence, The loss function of LightGBM is defined by Eq. (18):


(18) $$\begin{array}{l} \Gamma_{t}=\overset{n}{\underset{i=1}{\sum }}L\left(y_{i},F_{t-1}\left(x_{i}\right)+f_{t}\left(x_{i}\right)\right) \end{array}$$

The constant term in model (18) is removed for simplicity, and model (18) is transformed as Eq. (19):


(19) $$\begin{array}{l} \Gamma_{t}≅\overset{n}{\underset{i=1}{\sum }}\left(g_{i}f_{t}\left(x_{i}\right)+\frac{1}{2}h_{i}f_{t}^{2}\left(x_{i}\right)\right) \end{array}$$

where *g*_*i*_ and *h*_*i*_ denote the first-order and second-order derivatives of the loss function, respectively.

For a sample set, *I*_*j*_ related to leaf *j*, model (19) could be transformed as follows:


(20) $$\Gamma_{t}=\sum_{j=1}^{J}((\sum_{i\in I_{j}}g_{i})w_{j}+\frac{1}{2}(\sum_{i\in I_{j}}h_{i}+\lambda )w_{j}^{2})$$

Given a tree structure *q*(*x*), the optimal leaf weight $w_{j}^{*}$ of each leaf node and the maximum value of a scoring function Γ_*k*_ that evaluate the quality of *q*(*x*) are defined by Eqs. (21) and (22):


(21) $$\begin{array}{l} w_{j}^{*}=-\frac{\underset{i\in I_{j}}{\sum }g_{i}}{\underset{i\in I_{j}}{\sum }h_{i}+\lambda } \end{array}$$

(22) $$\begin{array}{l} \Gamma_{T}^{*}=-\frac{1}{2}\overset{J}{\underset{j=1}{\sum }}\frac{{\left(\sum_{i\in I_{j}}g_{i}\right)}^{2}}{\sum_{i\in I_{j}}h_{i}+\lambda } \end{array}$$

Consequently, the objective function is represented as Eq. (23):


(23) $$\begin{array}{l} G=\frac{1}{2}(\frac{{\left(\sum_{i\in I_{L}}g_{i}\right)}^{2}}{\sum_{i\in I_{L}}h_{i}+\lambda }+\frac{{\left(\sum_{i\in I_{R}}g_{i}\right)}^{2}}{\sum_{i\in I_{R}}h_{i}+\lambda }-\frac{{\left(\sum_{i\in I}g_{i}\right)}^{2}}{\sum_{i\in I}h_{i}+\lambda }) \end{array}$$

where *I*_*L*_ and *I*_*R*_ denote the example sets on the left and right sides, respectively.

## 3. Results

### 3.1. Experimental settings and evaluation metrics

Similar to RNMFMDA provided by Peng et al. (2020), the experiments were performed under three 5-fold cross validations (CVs) 20 times. For an MDA matrix *X*_*n*_, the three CVs were as follows:

- five-fold CV 1 (*CV*_1_): CV on diseases, i.e., in each round, 80% of *n*_*d*_ diseases in *X* was taken as training set and the remaining 20% was test set.
- five-fold CV 2 (*CV*_2_): CV on microbes, i.e., in each round, 80% of *n*_*m*_ microbes in *X* was taken as training set and the remaining 20% was test set.
- five-fold Cv 3 (*CV*_3_): CV on microbe–disease pairs, i.e., in each round, 80% of entries (microbe–disease pairs) in *X* were used as training set and the remaining 20% was test set.

In the sparse autoencoder, the neural network comprised an encoder and a decoder. The network structure was trained in Keras based on the TensorFlow backend. The structure comprised one input layer, three hidden layers, and an output layer. The number of each layer was 331, 256, 128, 96, and 64, respectively. The layers in the encoder and decoder were symmetric around the bottleneck. Tanh and ReLU were used as the activation functions in the output layer and the other layers, respectively. The optimization method used the Adam algorithm (Kingma and Ba, 2014). The batch size was set to 32 because a smaller batch size can make the model converge faster. The parameters λ, β, and ρ were set to 0.1, 0.0005, and 0.05, respectively. The final encoding size of the autoencoder is set to 64, that is, the features of MDAs were reduced to 64 dimensions.

For LightGBM, the parameters “num_leaves,” “learning_rate,” and “max_depth” denote the number of leaves in a tree, the speed of iteration, and the maximum depth of the tree, respectively. They were set to 31, 0.1, and –1, respectively. “Feature_fraction” and “bagging_fraction” are two hyperparameters in the optimization process. The former denotes the fraction of features at each iteration and was set to 0.9. The latter denotes the fraction of data and applies to boost the training and reduce overfitting. It was set to 0.9. “min_data” denotes the minimum number of records in a leaf and is also used to reduce overfitting. The parameters in the other four comparison methods were set to the defaults in corresponding publications. One microbe–disease pair is taken as a positive MDA when its association probability is greater than 50%, otherwise, it is taken as a negative MDA.

Four evaluation metrics were used to measure the performance of MDA prediction methods: accuracy, Matthews correlation coefficient (MCC) (Chicco and Jurman, 2020), area under the ROC curve (AUC), and area under the Precision-Recall curve (AUPR). Higher values for the four evaluation metrics represent better performance.

### 3.2. Performance comparison of SAELGMDA with the other four methods

To evaluate the performance of SAELGMDA, we compared it with four state-of-the-art MDA identification algorithms (MNNMDA, GATMDA, NTSHMDA, and LRLSHMDA) under three CVs on the HMDAD and Disbiome datasets, that is, *CV*_1_, *CV*_2_, and *CV*_3_.

#### 3.2.1. Performance comparison under *CV*_1_

**Figure 2:**
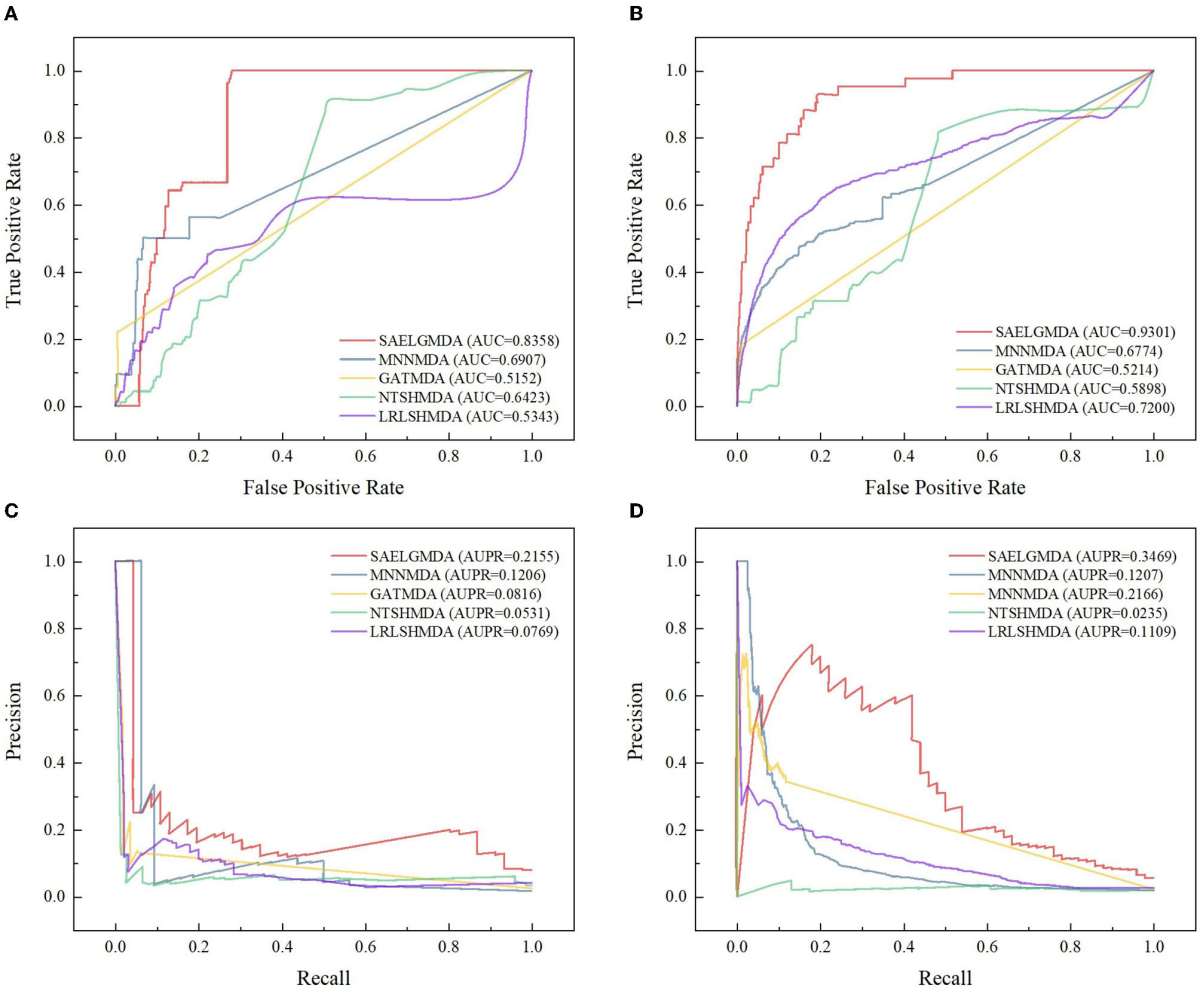
The ROC and the PR curves of five different methods under *CV*_1_ on the two databases. **(A, B)** Denote the ROC curves on the HMDAD and Disbiome databases, respectively. **(C, D)** Denote the PR curves on the HMDAD and Disbiome databases, respectively.

Table 1 shows accuracies, MCCs, AUCs, and AUPRs of SAELGMDA and the other four methods under *CV*_1_. The best performance in each column is described in Tables 1–**6**. As shown in Table 1, SAELGMDA computed the best MCC, AUC, and AUPR on the HMDAD database and the best accuracy, MCC, AUC, and AUPR on the Disbiome database, significantly outperforming the other four MDA prediction methods under *CV*_1_. Although accuracy was slightly less than MNNMDA and GATMDA on HMDAD, the difference was very tiny. Moreover, SAELGMDA outperformed the other methods, especially AUC and AUPR on the whole. In addition, although SAELGMDA outperformed the other four methods, all methods computed lower MCC and AUPR under *CV*_1_, which may be caused by fewer diseases. Figure 2 shows the ROC and PR curves of the five methods on the two databases under *CV*_1_.

**Table 1:** The performance of five MDA identification methods under *CV*_1_.

**Database**	**Method**	**Accuracy**	**MCC**	**AUC**	**AUPR**
HMDAD	SAELGMDA	0.9497 ± 0.0022	**0.1855** **±0.0116**	**0.8358** **±0.0109**	**0.2155** **±0.0075**
	MNNMDA	**0.9588** **±0.0009**	0.1085 ± 0.0109	0.6907 ± 0.0040	0.1206 ± 0.0021
	GATMDA	0.9562 ± 0.0009	0.0421 ± 0.0018	0.5152 ± 0.0003	0.0816 ± 0.0014
	NTSHMDA	0.9138 ± 0.0006	0.0101 ± 0.0008	0.6423 ± 0.0085	0.0531 ± 0.0007
	LRLSHMDA	0.9421 ± 0.0007	0.1182 ± 0.0028	0.5343 ± 0.0109	0.0769 ± 0.0006
Disbiome	SAELGMDA	**0.9819** **±0.0000**	**0.3431** **±0.0059**	**0.9301** **±0.0002**	**0.3469** **±0.0037**
	MNNMDA	0.9814 ± 0.0000	0.1521 ± 0.0008	0.6774 ± 0.0010	0.1207 ± 0.0004
	GATMDA	0.9807 ± 0.0000	0.0542 ± 0.0019	0.5214 ± 0.0005	0.2166 ± 0.0192
	NTSHMDA	0.9416 ± 0.0000	0.0204 ± 0.0000	0.5898 ± 0.0002	0.0235 ± 0.0000
	LRLSHMDA	0.9772 ± 0.0000	0.1469 ± 0.0004	0.7200 ± 0.0005	0.1109 ± 0.0002

#### 3.2.2. Performance comparison under *CV*_2_

**Figure 3:**
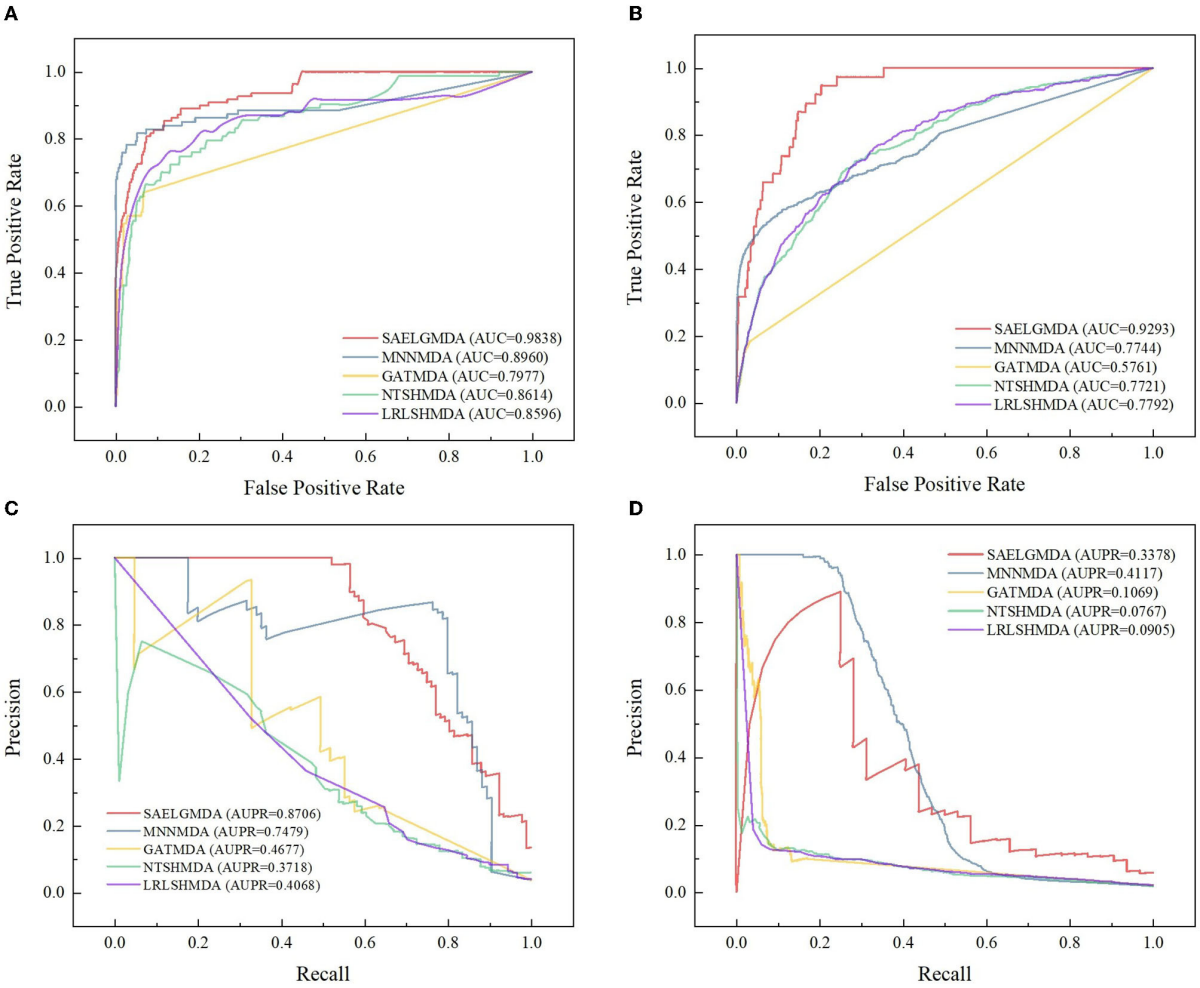
The ROC and the PR curves of five different methods under *CV*_2_ on the two databases. **(A, B)** Denote the ROC curves on the HMDAD and Disbiome databases, respectively. **(C, D)** Denote the PR curves on the HMDAD and Disbiome databases, respectively.

Table 2 demonstrates the prediction performance of SAELGMDA and the other four methods under *CV*_2_. The best performance in each column is described in boldface. As shown in Table 2, we observed that SAELGMDA computed the best accuracies, MCCs, and AUCs on the two databases under *CV*_2_. In particular, SAELGMDA obtained better MCC and AUPR on the HMDAD database than ones on the Disbiome database, which may be caused by different data structures. In addition, all five MDA prediction methods computed lower MCC and AUPR on the Disbiome database. Figure 3 shows the ROC and PR curves of the five methods under *CV*_2_.

**Table 2:** The performance of five MDA identification methods under *CV*_2_.

**Database**	**Method**	**Accuracy**	**MCC**	**AUC**	**AUPR**
HMDAD	SAELGMDA	**0.986** **±0.0000**	**0.8017** **±0.0017**	**0.9838** **±0.0001**	**0.8706** **±0.0010**
	MNNMDA	0.9654 ± 0.0000	0.344 ± 0.0034	0.896 ± 0.0016	0.7479 ± 0.0052
	GATMDA	0.9604 ± 0.0001	0.4775 ± 0.0065	0.7977 ± 0.0020	0.4677 ± 0.0096
	NTSHMDA	0.9642 ± 0.0000	0.4449 ± 0.0029	0.8614 ± 0.0007	0.3718 ± 0.0026
	LRLSHMDA	0.9642 ± 0.0000	0.4451 ± 0.0017	0.8596 ± 0.0009	0.4068 ± 0.0065
Disbiome	SAELGMDA	**0.9818** **±0.0000**	**0.3437** **±0.0040**	**0.9293** **±0.0003**	0.3378 ± 0.0049
	MNNMDA	0.9817 ± 0.0000	0.1907 ± 0.0016	0.7744 ± 0.0015	**0.4117** **±0.0023**
	GATMDA	0.9763 ± 0.0000	0.0915 ± 0.0011	0.5761 ± 0.0009	0.1069 ± 0.0031
	NTSHMDA	0.9723 ± 0.0000	0.0951 ± 0.0002	0.7721 ± 0.0002	0.0767 ± 0.0000
	LRLSHMDA	0.9657 ± 0.0000	0.1135 ± 0.0002	0.7792 ± 0.0002	0.0905 ± 0.0001

#### 3.2.3. Performance comparison under *CV*_3_

**Figure 4:**
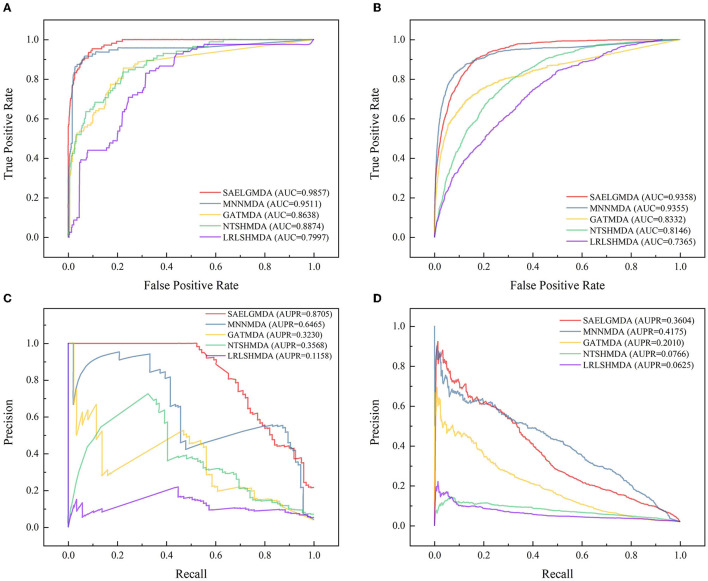
The ROC and the PR curves of five different methods under *CV*_3_ on the two databases. **(A, B)** Denote the ROC curves on the HMDAD and Disbiome databases, respectively. **(C, D)** Denote the PR curves on the HMDAD and Disbiome databases, respectively.

Table 3 shows the performance of SAELGMDA and the other four methods under *CV*_3_. The best performance in each column is described in boldface under *CV*_3_. The results from Table 3 suggest that SAELGMDA achieved the best accuracies, MCCs, and AUCs, significantly outperforming the other four MDA prediction methods under *CV*_3_. Moreover, the performance of all five methods under *CV*_3_ outperforms the ones under *CV*_1_ and *CV*_2_, demonstrating that more samples help improve the classification performance. Figure 4 shows the ROC and PR curves of the five methods under *CV*_3_.

**Table 3:** The performance of five MDA identification methods under *CV*_3_.

**Database**	**Method**	**Accuracy**	**MCC**	**AUC**	**AUPR**
HMDAD	SAELGMDA	**0.9859** **±0.0000**	**0.7978** **±0.0010**	**0.9857** **±0.0000**	**0.8705** **±0.0008**
	MNNMDA	0.9653 ± 0.0000	0.3401 ± 0.0055	0.9511 ± 0.0002	0.6465 ± 0.0023
	GATMDA	0.8935 ± 0.0004	0.3427 ± 0.0020	0.8638 ± 0.0007	0.3230 ± 0.0060
	NTSHMDA	0.9613 ± 0.0000	0.1783 ± 0.0338	0.8874 ± 0.0003	0.3568 ± 0.0026
	LRLSHMDA	0.9453 ± 0.0000	0.0568 ± 0.0011	0.7997 ± 0.0002	0.1158 ± 0.0002
Disbiome	SAELGMDA	**0.9826** **±0.0000**	**0.3376** **±0.0004**	**0.9358** **±0.0000**	0.3604 ± 0.0004
	MNNMDA	0.9815 ± 0.0000	0.1523 ± 0.0012	0.9355 ± 0.0000	**0.4175** **±0.0002**
	GATMDA	0.8461 ± 0.0004	0.2032 ± 0.0002	0.8332 ± 0.0001	0.201 ± 0.0004
	NTSHMDA	0.9807 ± 0.0000	0.0207 ± 0.0002	0.8146 ± 0.0000	0.0766 ± 0.0000
	LRLSHMDA	0.9781 ± 0.0000	0.0744 ± 0.0002	0.7365 ± 0.0000	0.0625 ± 0.0000

#### 3.2.4. Performance comparison of LightGBM and two classification models

To measure the MDA classification performance of LightGBM, we compared it with two classical boosting algorithms, XGBoost and NGBoost. Extreme Gradient Boosting (XGBoost) is an ensemble learning method based on a gradient boost tree and can accurately cope with multicollinearity impact and complicated non-linearity interactions (Chen and Guestrin, 2016; Zhu and Zhu, 2019). Natural Gradient Boosting (NGBoost) uses natural gradients instead of regular gradients to implement flexible probabilistic forecast (Duan et al., 2020). Tables 4–6 show the accuracy, MCC, AUC, and AUPR of LightGBM, NGBoost, and XGBoost on the Disbiome and HMDAD datasets under three cross validations. The results from Tables 4–6 indicate that LightGBM obtained better performance on the majority of conditions and can be used to improve MDA classification ability.

**Table 4:** The performance of three classification models under *CV*_1_.

**Database**	**Method**	**Accuracy**	**MCC**	**AUC**	**AUPR**
HMDAD	LightGBM	0.9497 ± 0.0022	**0.1855** **±0.0116**	0.8358 ± 0.0109	**0.2155** **±0.0075**
	NGBoost	**0.9526** **±0.0016**	0.1728 ± 0.0107	0.8301 ± 0.0097	0.1988 ± 0.0056
	XGBoost	0.946 ± 0.0018	0.1832 ± 0.0092	**0.8385** **±0.0051**	0.1843 ± 0.0050
Disbiome	LightGBM	0.9819 ± 0.0000	0.3431 ± 0.0059	**0.9301** **±0.0002**	0.3469 ± 0.0037
	NGBoost	**0.9826** **±0.0000**	**0.3631** **±0.0032**	0.9284 ± 0.0002	**0.3598** **±0.0027**
	XGBoost	0.9775 ± 0.0000	0.2706 ± 0.0034	0.905 ± 0.0003	0.2494 ± 0.002

**Table 5:** The performance of three classification models under *CV*_2_.

**Database**	**Method**	**Accuracy**	**MCC**	**AUC**	**AUPR**
HMDAD	LightGBM	**0.986** **±0.0000**	**0.8017** **±0.0017**	**0.9838** **±0.0001**	**0.8706** **±0.0010**
	NGBoost	0.9854 ± 0.0046	0.794 ± 0.0511	0.9808 ± 0.0102	0.8615 ± 0.0447
	XGBoost	0.9846 ± 0.0000	0.7814 ± 0.0027	0.9803 ± 0.0001	0.8434 ± 0.0021
Disbiome	LightGBM	**0.9818** **±0.0000**	**0.3437** **±0.0040**	**0.9293** **±0.0003**	0.3378 ± 0.0049
	NGBoost	0.9817 ± 0.0034	0.3382 ± 0.0756	0.9284 ± 0.0164	**0.3597** **±0.0920**
	XGBoost	0.9771 ± 0.0054	0.2671 ± 0.0619	0.904 ± 0.0186	0.2502 ± 0.0640

**Table 6:** The performance of three classification models under *CV*_3_.

**Database**	**Method**	**Accuracy**	**MCC**	**AUC**	**AUPR**
HMDAD	LightGBM	**0.9859** **±0.0000**	**0.7978** **±0.0010**	**0.9857** **±0.0000**	**0.8705** **±0.0008**
	NGBoost	0.9854 ± 0.0000	0.7905 ± 0.0013	0.9821 ± 0.0000	0.8625 ± 0.0013
	XGBoost	0.9838 ± 0.0000	0.7679 ± 0.0011	0.9804 ± 0.0000	0.835 ± 0.0010
Disbiome	SAELGMDA	**0.9826** **±0.0000**	0.3376 ± 0.0004	**0.9358** **±0.0000**	0.3604 ± 0.0004
	LightGBM	**0.9826** **±0.0000**	**0.3396** **±0.0003**	0.9336 ± 0.0000	**0.3764** **±0.0002**
	XGBoost	0.9805 ± 0.0000	0.2375 ± 0.0039	0.9129 ± 0.0000	0.2594 ± 0.0002

#### 3.2.5. Computational time analysis

**Figure 5:**
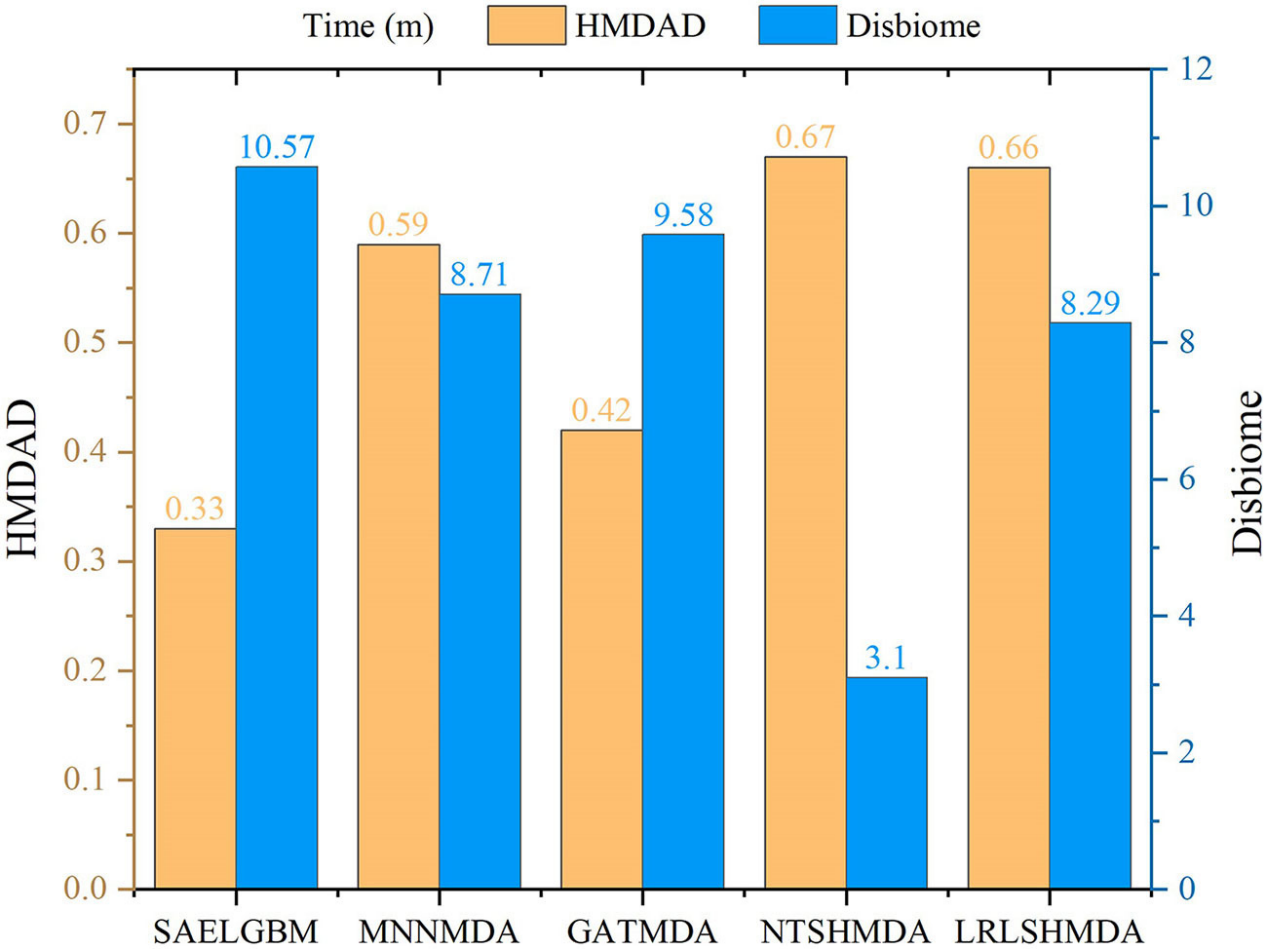
Computational time of five MDA methods.

We compared the computational time of SAELGMDA with the other four MDA prediction models, MNNMDA, GATMDA, NTSHMDA, and LRLSHMDA. The experiments were run on a machine with an AMD EPYC 7302 CPU, a GeForce RTX 2080 Ti, and 256GB RAM on Ubuntu 20.04.4 LTS operating system. Figure 5 shows computational time (m) of the five MDA prediction models on five-fold cross validation for one time on two MDA datasets. As shown in Figure 5, SAELGMDA is the most rapid method on the HMDAD dataset and the slowest one on the Disbiome dataset. SAELGMDA need only to spend 10.57 min, although it run slowly on the Disbiome database. In summary, SAELGMDA need not too much time on the two MDA datasets.

### 3.3. Case study

In this section, we predicted potential MDAs on the two MDA databases. In addition, multiple evidence suggests that colorectal cancer, inflammatory bowel diseases, and lung cancer have dense linkages with microbes (Guarner and Malagelada, 2003; Müller and Macpherson, 2006; Zhang et al., 2015; Mármol et al., 2017; Chicco and Jurman, 2020). In this section, we aim to find possible microbes for the three diseases using the proposed SAELGMDA method. For the three diseases, microbes that are known to associate with them were removed. Next, we computed the association scores between them and all microbes. Third, the computed scores were sorted in descending order. Finally, the top 20 microbes with the highest association scores with them were listed and confirmed by the existing publications.

#### 3.3.1. Finding new MDAs based on known MDAs

**Figure 6:**
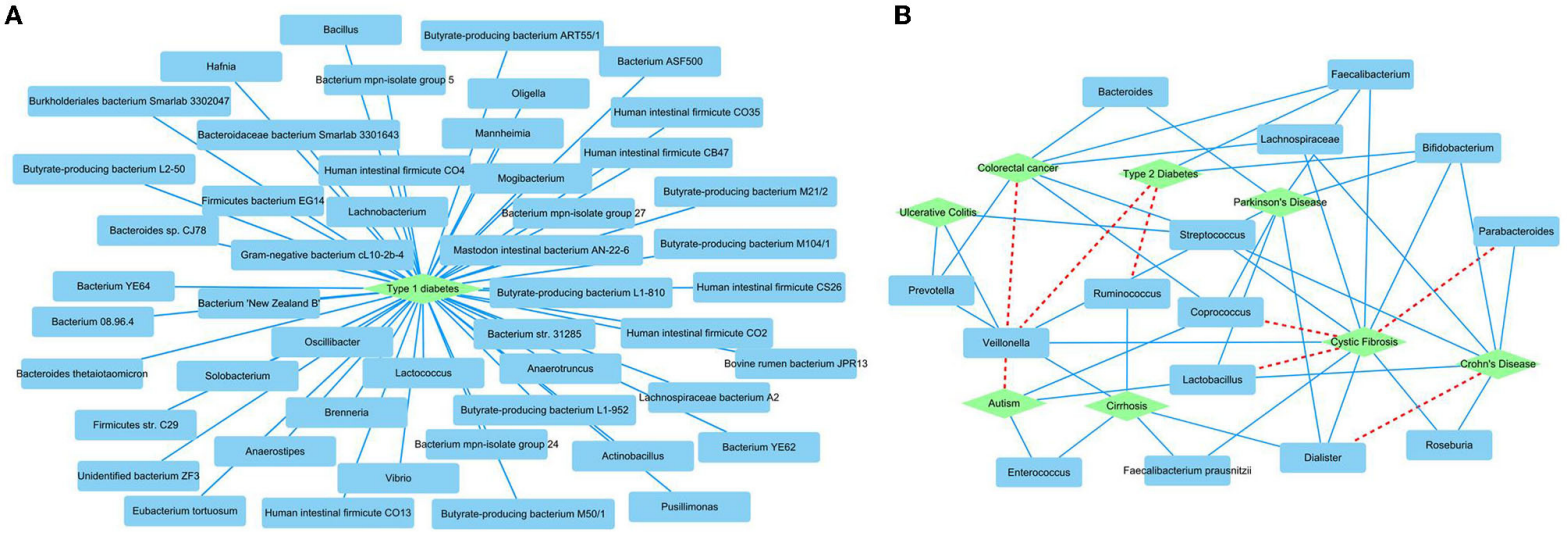
The predicted top 50 MDAs on the HMDAD **(A)** and Disbiome **(B)** databases.

We further predicted new MDAs based on known MDAs using SAELGMDA. The predicted top 50 MDAs are shown in Figure 6. In Figure 6, sky blue solid lines and red dotted lines represent known and unknown MDAs obtained from SAELGMDA, respectively. Deep sky blue round rectangles represent microbes and green diamonds denote diseases.

On the HMDAD database, all predicted top 50 MDAs have been known to be associated with the database. SAELGMDA predicted that Actinobacteria and liver cirrhosis have the highest association probability with the ranking of 130 among all 11,388 microbe–disease pairs. Actinobacteria have been reported to associate with liver disease (Bull-Otterson et al., 2013). The expansion of Proteobacteria and Actinobacteria has a pathogenic effect on alcoholic liver disease (Bull-Otterson et al., 2013).

In the Disbiome database, SAELGMDA predicted that *Veillonella* may associate with autism with a ranking of three among all 229,336 microbe–disease pairs. Zhang et al. (2018) has reported that the abundance of *Veillonella* was severely decreased in stools of children suffering from autism spectrum disorder. The decreasing of its abundance has been also found in subjects involved in autism (Strati et al., 2017). Furthermore, the decreased *Veillonella* may affect the fermentation of lactate in the autism children (Gronow et al., 2010).

#### 3.3.2. Colorectal cancer-related microbe identification

Colorectal cancer is the third most frequent cause of cancer mortality worldwide, severely threatening global life and health (Biller and Schrag, 2021; Saeed et al., 2021; Wong et al., 2023). There are more than 1.85 million colorectal cancer cases and 850,000 colorectal cancer-related deaths each year. In total, 20% of patients with colorectal cancer have metastasis cancer among new colorectal cancer diagnoses. It has been reported that ~70%–75% of patients survive more than 1 year, 30%–35% more than 3 years, and fewer than 20% more than 5 years among patients diagnosed with metastatic colorectal cancer. Although colonoscopy has been widely applied to the screen, its effect on colorectal cancer remains unclear (Bretthauer et al., 2022). Table 7 shows the top 20 microbes associated with colorectal cancer on the HMDAD database.

**Table 7:** The top 20 microbes related to colorectal cancer inferred by SAELGMDA on the HMDAD database.

**Rank**	**Microbe**	**Evidence**
1	*Fusobacterium nucleatum*	Confirmed by HMDAD
2	Firmicutes	Confirmed by HMDAD
3	Proteobacteria	PMID: 24 603 888, 27 194 068, 32 298 987
4	*Prevotella*	Confirmed by HMDAD
5	*Bacteroidetes*	Confirmed by HMDAD
6	Clostridia	Confirmed by HMDAD
7	*Fusobacterium*	Confirmed by HMDAD
8	*Bacteroides*	Confirmed by HMDAD
9	*Pseudomonas*	PMID: 33 998 814, 25699023, 25 217 106
10	*Haemophilus*	PMID: 31 358 825, 26 549 775
11	Actinobacteria	PMID: 35 899 111, 35 049 922
12	*Acinetobacter*	PMID: 32 738 757, 32 595 614
13	*Corynebacterium*	PMID: 313 873, 646 934
14	*Lactobacillus*	PMID: 36 162 222, 22 830 611, 35 808 840
15	*Streptococcus*	PMID: 9 771 449, 21 960 713, 21 247 505, 18 990 738, 16 845 563
16	*Clostridium difficile*	PMID: 26 691 472, 28 060 753, 21 152 135, 1 626 323
17	*Faecalibacterium prausnitzii*	PMID: 26 595 550, 35 625 865, 32 675 782
18	*Clostridium coccoides*	Unconfirmed
19	Lachnospiraceae	PMID: 28 988 196, 36 893 736
20	*Helicobacter pylori*	PMID: 22 294 430, 16 579 836, 18 506 454, 31 393 968

For colorectal cancer, as shown in Table 7, 19 microbes have been confirmed to have associations with colorectal cancer by the existing literature on the top 20 inferred microbes on the HMDAD database. For example, pseudomonas is distinctly less abundant in cancer tissues than normal tissues and has been increasingly taken as an emerging clinic-related opportunistic pathogen (Decker and Palmore, 2014; Gao et al., 2015). *Haemophilus parainfluenzae* demonstrates higher representation in colorectal cancer subjects but is scarcely investigated in control subjects (Kasai et al., 2016). Research in 219 patients with colorectal cancer has suggested that clostridium difficile has a dense relationship with colorectal cancer (Yeom et al., 2010). Helicobacter pylori infection has been reported to be a potential risk increase factor of left-sided colorectal cancer (Zhang et al., 2012).

Moreover, we inferred that *Clostridium coccoides* has a possible association with colorectal cancer. *Clostridium coccoides* is taken as one of the most prevalent groups of bacteria in human intestines. They constitute ~60% of mucin-adhered microbiota and comprise different species with high oxygen-sensitive anaerobes (such as *Clostridium, Coprococcus, Eubacterium*, and *Ruminococcus*). They contribute to the prevention of colonization of vancomycin-resistant *Enterococcus* in an antibiotic-treated mouse model (Grenda et al., 2022). The association between *Clostridium coccoides* and colorectal cancer needs further validation.

#### 3.3.3. Inflammatory bowel disease-related microbe identification

Inflammatory bowel disease is one of the idiopathic inflammatory bowel disorders that severely affect the gastrointestinal tract. It has become a global, chronic, and life-threatening disease over the last few decades. Mak et al. (2020) predicted that patients with inflammatory bowel disease may be an exponential increase worldwide. It typically includes Crohn's disease and ulcerative colitis. It manifests progressive and unpredictable features and is partially caused by bacteria that activate patient's immune system to protect against foreign substances (Lomax et al., 2006; Kaplan and Windsor, 2021). It has a close relationship with microbes. Identification of associated microbes for the disease helps us better equip to stem its global rise in future. Table 8 lists the top 20 microbes associated with the disease on the HMDAD database.

**Table 8:** The top 20 microbes related to inflammatory bowel disease inferred by SAELGMDA on the HMDAD database.

**Rank**	**Microbe**	**Evidence**
1	*Bacteroidetes*	PMID: 12 906 096, 27 999 802, 21 575 910
2	Proteobacteria	Confirmed by HMDAD
3	Firmicutes	PMID: 19 235 886
4	Lachnospiraceae	Confirmed by HMDAD
5	*Haemophilus*	PMID: 33 666 710, 30 685 379
6	Actinobacteria	Confirmed by HMDAD
7	*Prevotella*	PMID: 28 542 929, 26 468 751
8	*Clostridium coccoides*	PMID: 27 687 331, 16 432 374
9	*Bifidobacterium*	PMID: 34 337 079, 25 793 197, 24 478 468, 25 391 346
10	*Lactobacillus*	PMID: 29 854 599, 32 509 162, 15 664 933
11	*Staphylococcus aureus*	PMID: 31 698 044
12	*Fusobacterium*	PMID: 27 139 617, 33 996 366, 25 576 662
13	Clostridia	PMID: 22 508 484, 28 506 071
14	*Clostridium difficile*	PMID: 22 508 484, 28 506 071
15	*Helicobacter pylori*	PMID: 24 914 359, 19 760 778
16	*Streptococcus*	PMID: 30 392 911, 23 679 203, 28 618 865, 16 868 828
17	*Bacteroides vulgatus*	PMID: 12 906 096, 12 162 408
18	*Bacteroides*	PMID: 12 906 096, 12 162 408
19	Oxalobacteraceae	PMID: 29228248
20	Sphingomonadaceae	Unconfirmed

As shown in Table 8, 19 microbes have been validated to link to inflammatory bowel disorders by existing literature on the predicted top 20 microbes associated with it on the HMDAD database. Researchers reported that Firmicutes were less represented in patients suffered from inflammatory bowel disease than healthy subjects (Sokol et al., 2009). *Streptococcus* and *Haemophilus* were highly represented in patients with inflammatory bowel disease (Heidarian et al., 2019). *Prevotella* was reduced in pediatric Crohn's disease (Lewis et al., 2015). *Clostridium coccoides* was less abundant in patients with active inflammatory bowel disease than ones in remission (Prosberg et al., 2016).

In addition, we predicted that Sphingomonadaceae dense links to inflammatory bowel disease. Sphingomonadaceae family has high abundance in marine waters, freshwater, and even drinking water. They can degrade lignin-derived compounds and refractory organic matter that comprise monocyclic and polycyclic aromatic hydrocarbons (Shen S. et al., 2022). Sphingomonadaceae are significantly accommodated to bile salts through metabolic pathways (de Vries et al., 2019). In addition, Sphingomonadaceae has a high linkage with triclosan degradation in nitrification and denitrification systems (Dai et al., 2022). Microbial communities were adapted to Bisphenol A through the selection of Sphingomonadaceae populations including *Sphingobium, Novosphingobium*, and *Sphingopyxis*. The selected Sphingomonadaceae for Bisphenol A demonstrated higher Bisphenol A metabolic activity (Oh and Choi, 2019). The association between Sphingomonadaceae and inflammatory bowel disease needs further validation.

#### 3.3.4. Lung cancer-related microbe identification

Lung cancer is one of the leading causes of cancer-related deaths worldwide. It accounts for ~18% of global cancer deaths (Sung et al., 2021). More than 350 patients died from lung cancer each day in the United States (Siegel et al., 2022). It has the highest incidence and mortality compared with other cancer types in China (Xia et al., 2022). We used the proposed SAELGMDA model to identify potential microbes for lung cancer. Table 9 lists the top 20 microbes associated with it on the Disbiome database. As shown in Table 9, all 20 top microbes have been confirmed to be associated with lung cancer by existing literatures or the Disbiome database. The results again validated the MDA prediction performance of SAELGMDA.

**Table 9:** The top 20 microbes associated with lung cancer identified by SAELGMDA on the Disbiome database.

**Rank**	**Microbe**	**Evidence**
1	*Acidovorax*	Confirmed by Disbiome
2	*Parabacteroides*	PMID: 30 693 820, 32 010 563, 33 302 682, 33 302 682, 32 329 229, 30 693 820
3	*Diaphorobacter*	Confirmed by Disbiome
4	*Bifidobacterium*	Confirmed by Disbiome
5	*Roseburia*	PMID: 33 302 682, 32 227 387, 35 735 103
6	*Bacteroides*	PMID: 306 938 20, 36 498 063, 30 416 658,
7	*Lactobacillus*	PMID: 26 125 762, 36 361 537, 36 638 662
8	*Leptotrichia*	PMID: 34 432 217, 33 454 779
9	*Prevotella*	Confirmed by Disbiome
10	*Enterococcus*	PMID: 33 302 682, 27 717 798, 31 065 547, 33 111 503
11	*Streptococcus*	Confirmed by Disbiome
12	*Corynebacterium*	PMID: 350 388, 6 362 846, 6 998 933, 6 318 791
13	*Porphyromonas*	PMID: 33 279 803,32 615 270
14	*Alistipes*	PMID: 33 939 976, 34 793 492, 35 115 705
15	*Haemophilus*	PMID: 21 407 824, 21 407 824, 27 052 615, 21 098 042, 34 963 470
16	*Klebsiella*	PMID: 32 099 416, 24 706 703
17	*Dialister*	PMID: 30 416 658, 29 023 689, 34 063 829, 31 595 156
18	*Ruminococcus*	PMID: 32 227 387, 33 302 682, 36 737 654, 33 603 241, 32 240 032
19	*Pseudomonas*	PMID: 27 507 537, 25 801 231, 30 101 407
20	*Escherichia*	PMID: 18 496 688, 10.1158/1538-7445.AM2023-5185

### 3.4. Discussion and conclusion

Systematic identification of associations between microbes and diseases significantly contributes to the understanding of the complex pathogenic mechanism of various diseases (Takahashi et al., 2018; Zhou et al., 2018; Yang et al., 2022). In particular, computational pathogenic microorganism discovery helps to capture potential biomarkers from candidate compounds for human complex diseases (Barrows et al., 2016; Zhu et al., 2021).

Here, we developed a computational method called SAELGMDA to improve MDA prediction. First, microbe similarity and disease similarity were computed via their function similarity and GIPK similarity. Second, one microbe–disease pair was represented as a feature vector based on microbe similarity matrix and disease similarity matrix. Third, the obtained high-dimensional features were mapped to a low-dimensional space based on a sparse autoencoder. Finally, unknown microbe–disease pairs were classified using LightGBM.

Our proposed SAELGMDA method was compared with MNNMDA, GATMDA, LRLSHMDA, and NTSHMDA. Experimental results under *CV*_1_, *CV*_2_, and *CV*_3_ show that SAELGMDA outperforms the above four methods. SAELGMDA obtains the superior MDA identification ability. To investigate the MDA classification performance of LightGBM, we further compared it with XGBoost and NGBoost. The results demonstrate that LightGBM obtained better accuracy. Case studies demonstrate that there are possible associations between *Clostridium coccoides* and colorectal cancer, between Sphingomonadaceae and inflammatory bowel disease, and between *Veillonella* and autism and needs further validation.

We used two MDA databases (Disbiome and HMDAD) to investigate the performance of our proposed SAELGMDA method. The HMDAD dataset is a small dataset and Disbiome is a larger dataset. Under *CV*_1_, the performance of SAELGMDA, GATMDA, and LRLSHMDA on the Disbiome dataset outperforms the ones on the HMDAD dataset, demonstrating more data contribute to the performance improvement for the three methods under *CV*_1_. Under *CV*_2_ and *CV*_3_, all five methods computed higher accuracy and AUC on the two datasets. However, MCC and AUPR computed by these five methods significantly decreased the Disbiome dataset compared with the HMDAD dataset. It may be caused by data imbalance; that is, the generalization ability of SAELGMDA is good when identifying potential associated microbes for a query disease. However, its generalization ability needs further improvement under *CV*_2_ and *CV*_3_.

Although SAELGMDA outperformed the other four methods under the majority of condition on the HMDAD and Disbiome databases, the performance of all five MDA prediction methods, especially MCC and AUPR, remains an improvement. In future, we will integrate more biological data, such as microbe–drug associations and disease–gene associations, to extract effective features for microbe–disease pairs. Furthermore, we will explore new dimensional reduction algorithms and classification models to improve MDA prediction by combining deep learning.

## Data availability statement

The original contributions presented in the study are included in the article/supplementary material, further inquiries can be directed to the corresponding authors.

## Author contributions

FW and HY: conceptualization and validation. LP: funding acquisition. YW, LP, and XL: project administration. FW: writing—original draft and software. HY, LP, and XL: writing—reviewing and editing and investigation. FW and LP: methodology. All authors contributed to the article and approved the submitted version.
